# Mean Birth Weight and Mean placental Weight among Deliveries in a Tertiary Care Hospital

**DOI:** 10.31729/jnma.3660

**Published:** 2019-10-31

**Authors:** Raju Kafle, Kabiraj Nibedita, Binod Kumar Gupta

**Affiliations:** 1 Department of Paediatrics, Universal College of Medical Sciences, Bhairahawa, Nepal

**Keywords:** *birth weight*, *maternal nutrition*, *placenta.*

## Abstract

**Introduction:**

Placenta is an organ that connects the developing fetus to the uterine wall to allow nutrient uptake, provide thermo-regulation to the fetus, waste elimination, and gas exchange. The present study was undertaken to look for mean birth weight and placental weight among deliveries in a tertiary care hospital.

**Methods:**

A descriptive cross-sectional study was conducted in a tertiary hospital of Nepal. Ethical clearance was taken from institutional review comittee of hospital. Mothers with term and preterm gestation, their infants and the placentas were the subjects for the study. The study was conducted on 158 term and preterm deliveries. Placental weight, birth weight, gestational age, neonates’ gender, weight, length and head circumference were recorded.

**Results:**

The mean of weight of total 158 placentas was 449.24±82.07 g and the mean of birth weights was 2872.84±478.88 g. Out of 158 deliveries, 138 (87.4%) babies were of term gestation and 20 (12.6%) babies were preterm.

**Conclusions:**

Mean birth weight and placental weights are similar to that found on similar studies done on other hospitals. Knowing the mean placental weight and birth weight which relates to different functional dimensions of placenta and baby growth helps for further evaluation of placenta and baby.

## INTRODUCTION

Placenta plays a vital role in normal fetal development and failure of placenta to gain weight and insufficiency of its function can result in fetal disorders. It has been shown that placental weight has a significant role in fetal growth in terms of weight, body length, and cord length but it has no significant role in the presence of meconium-stained fluid.^1^ While some other studies have shown less correlation between above mentioned factors and placental weights.^2^

The placenta is attractive because of its availability and the ease with which it is measured and is also a cost-effective measure to correlate certain important aspects of health and disease. In Nepal, with the national efforts for “Safe motherhood Program”, studies are essential on the pattern of fetal and placental growth. Also, the data on birth weight and placental weight are not available in context of Nepal.

The aim of this study was to look for mean birth weight and mean placental weight among deliveries in a tertiary care hospital of women in Bhairahawa Nepal.

## METHODS

It is a descriptive cross-sectional study carried out among mothers with term and preterm gestation, and their infants at Universal College of Medical Sciences (UCMS), Teaching Hospital, Bhairahawa, Nepal from Jan 2012 to November 2012. Ethical clearance was taken from institutional review comittee of UCMS Teaching Hospital. Informed consent was obtained from all participants. All women with singleton pregnancy and all term, preterm and post term gestation delivered at births were included in the study. Intrauterine fetal death, women with unknown/ disputed gestational age, women with medical illnesses, pregnancy induced hypertension, eclampsia and preeclampsia, Neonates with congenital anomalies, women with twin/multiple pregnancy were excluded from the study. Placental weight, mean birth weight, gestational age, neonates’ gender, weight, length and head circumference were recorded. Maternal age, weight, height were also noted and haemoglobin was estimated. The placental weight ratio, Ponderal index and maternal body mass index were calculated. These values were thus entered in a structured proforma.

Data collection was done by filling self-structured performa designed for the study. Data was collected throughout the study period. Convenience sampling was done and sample size was calculated using following formula:

n= Z^2^sd^2^/e^2^

where,

n= minimum sample size
Z= for Confidence Interval of 90 %
Sd= standard deviation (1) from the pilot study done on 10% of the total sample size calculated.
e= maximum allowable error=13%
calculated sample size=158

Sample size of 158 was calculated. Data was analyzed using SPSS package.

## RESULTS

Out of 158 deliveries, 138 (87.4%) babies were of term gestation with mean placental weights of 455.76 ±79.75 g and mean birth weights of 2939.93±431.82 g and 20 (12.6%) babies were preterm with mean placental weights of 405.25±85.75 g and mean birth weights of 2410±540.61 g. Female: male ratio was 1.6. Mean and standard deviation of placental weights of term and preterm babies and mean and standard deviation of birth weights of term and preterm babies are observed (Table 1 and 2). The ratio of placental weight to birth weight was 1:6.6. The mean of placental weight was 15.85±2.87 and the mean of birth weight/birth length ratio was 59.65±8.83. Percentiles for birth weights in different placental weight groups are calculated (Table 3).

**Table 1:** Mean and standard deviation of placental weights of term and preterm babies.

	Mean	S.D
Term (n = 138)	455.76	79.75
Preterm (n = 20)	405.25	85.75

**Table 2:** Mean and standard deviation of birth weights of term and preterm babies.

	Mean	S.D
Term (n = 138)	2939.93	431.82
Preterm (n = 20)	2410.00	540.61

**Table 3:** Percentiles for birth weights in different placental weight groups.

Corresponding Percentiles of birth weights	Placental weight groups		
	300-400	401-500	501-600
	(n=22)	(n=87)	(n=36)
3^rd^	1694.5	2179	2505
10^th^	1765	2500	2600
50^th^	2500	2900	3250
90th	2980	3400	3500

## DISCUSSION

The mean placental weight of total 158 placentas in the present study was similar to the placental weights carried out by Pachauri et al which was 406.3± 83.09 g.^3^ However, the value was less than the value 529.7±113g, obtained by Asgharnia et al in Iran.^4^ The variations in the mean weight of the placenta may be due to variations in the methodology of preparing and weighing the placentas and may also be due to differences in nutritional status of mothers in different parts of world. Similar to our study, Heinonen et al. showed that placental actual weight was lower in Small for Gestational Age (SGA) infants than in Appropriate for Gestational Age (AGA) infants with the same birth weight.^5^ It seems that low birth weight should be related to low functional tissue mass of placenta; and this is accompanied by diminution of the area for exchange between mother and fetus, both at the villi and at fetal capillary surface area. Thus, the ability of exchanging oxygen and nutrition from mother to fetus is curtailed.

The mean gestational age of the babies was 38.2±2.7 weeks at birth. The infants’ gestational age at birth was calculated from the last normal menstrual period. This is also similar to the recorded gestational age of the babies at delivery, using the Dubowitz method of estimation of gestational age, in an earlier study in Jos, which was 38.63±1.25 weeks.^6^

The mean birth weight of the babies delivered during the period of review was 2939.93±431.82g. This figure is higher than the mean birth weight 2.89±0.47 kg in an earlier study in 1999 in Jos^6^ and lower to the 3.167 ±0.45 recorded in Ilorin.^7^ The slight lower birth weight compared with that in Ilorin might be due to the higher altitude of that area where studied was done. The birth weight in this study was higher than that reported from Nnewi, Anambra state birth weight 2.89 kg.^8^ The average birth weight of babies in an Indian study was reported as 2.8 kg.^9^ This is also lower than that in this study. The mean birth weight of the infants was higher in males compared with female babies, which is in agreement with reports from other centres.^6,7^

## CONCLUSIONS

Mean birth weight and placental weight are similar to that found on similar studies done. Further the placental weight is dependent on maternal nutritional state. It has also been observed that placental actual weight was lower in SGA infants than in AGA infants with the same birth weight. Thus, weight of the placenta is amongst the simplest parameters that can be recorded easily and there can be the relation with pregnancy abnormality which is of great interest to clinicians.

## Conflict of Interest:

None.
